# Comparative Proteomic Analysis of Differentially Expressed Proteins in the Urine of Reservoir Hosts of Leptospirosis

**DOI:** 10.1371/journal.pone.0026046

**Published:** 2011-10-17

**Authors:** Jarlath E. Nally, Avril M. Monahan, Ian S. Miller, Ruben Bonilla-Santiago, Puneet Souda, Julian P. Whitelegge

**Affiliations:** 1 Veterinary Science Centre, UCD School of Agriculture Food Science and Veterinary Medicine, University College Dublin, Belfield, Dublin, Republic of Ireland; 2 UCD Conway Institute of Biomolecular and Biomedical Research, College of Life Sciences, University College Dublin, Belfield, Dublin, Republic of Ireland; 3 The Pasarow Mass Spectrometry Laboratory, Departments of Psychiatry and Biobehavioral Sciences, Chemistry and Biochemistry, and the Neuropsychiatric Institute, University of California, Los Angeles, California, United States of America; Instituto Butantan, Brazil

## Abstract

*Rattus norvegicus* is a natural reservoir host for pathogenic species of *Leptospira*. Experimentally infected rats remain clinically normal, yet persistently excrete large numbers of leptospires from colonized renal tubules via urine, despite a specific host immune response. Whilst persistent renal colonization and shedding is facilitated in part by differential antigen expression by leptospires to evade host immune responses, there is limited understanding of kidney and urinary proteins expressed by the host that facilitates such biological equilibrium. Urine pellets were collected from experimentally infected rats shedding leptospires and compared to urine from non-infected controls spiked with in vitro cultivated leptospires for analysis by 2-D DIGE. Differentially expressed host proteins include membrane metallo endopeptidase, napsin A aspartic peptidase, vacuolar H+ATPase, kidney aminopeptidase and immunoglobulin G and A. Loa22, a virulence factor of *Leptospira*, as well as the GroEL, were increased in leptospires excreted in urine compared to in vitro cultivated leptospires. Urinary IgG from infected rats was specific for leptospires. Results confirm differential protein expression by both host and pathogen during chronic disease and include markers of kidney function and immunoglobulin which are potential biomarkers of infection.

## Introduction

The laboratory rat is an indispensable tool in experimental medicine and is used extensively as a model organism for studying human normal and disease processes. However, the rat is also a natural reservoir host for many infectious agents, including pathogenic species of *Leptospira* [1]. Reservoir hosts of leptospirosis are typically asymptomatic, often serologically negative, and include a range of mammalian host species such as rats, dogs and cattle [2], [3], [4]. More recently, a cohort of Peruvian women were identified as asymptomatic carriers of leptospires [5]. Pathogenic species of *Leptospira* colonize the renal tubules of reservoir hosts, from which they are shed via urine into the environment in which they can survive in suitable moist, slightly alkaline conditions. Contact with contaminated water sources, or directly with contaminated urine, can result in infection in incidental hosts such as humans since leptospires can penetrate breaches of the skin, or mucosal surfaces such as conjunctival tissue.

The complex interplay of host and pathogen has evolved over millennia, with pathogens evolving systems that allow a spectrum of conditions such as chronic, persistent carriage in some hosts, compared to acute, fulminant infection in others. Whilst the significance of the rat as a carrier and reservoir host of pathogenic *Leptospira* species was first described in 1917, there have been limited studies using the rat to elucidate the molecular basis of this unique host-pathogen biological equilibrium [6]. Five days after experimental infection, there is a rapid clearance of leptospires from all rat tissues except kidney [7]. Experimentally infected *Rattus norvegicus* appear clinically normal yet excrete large numbers of leptospires (up to >10^6^/ml) in urine, despite a specific host immune response [8]. Persistent infection and shedding from colonized renal tubules is facilitated in part by the ability of leptospires to evade specific antibody responses by differential antigen expression [8].

Chronically infected rats are often the primary reservoir host of infection for transmission of leptospirosis to human patients, causing acute severe disease processes [9], [10]. Given the importance of rat-borne transmission of leptospirosis via urine, and the use of the rat model of chronic leptospirosis to emulate persistent asymptomatic carriage in a range of mammalian host species including humans, a proteomic analysis was performed on urine of experimentally infected rats compared to non-infected controls by 2-D DIGE. It was hypothesized that infected rats modulate expression of kidney and urinary proteins during persistent renal colonization and excretion of leptospires into the environment, the identification of which can facilitate a better understanding of pathogenic mechanisms of chronic leptospirosis, the host response to infection and the potential for the identification of novel biomarkers of chronic infection.

## Methods

### Ethics Statement

All animal protocols were approved according to the Cruelty to Animals Act, 1876, as amended by European Communities (Amendment to cruelty to Animals Act 1879) Regulations 2002 and 2005. Animal protocols in this study were approved by the University College Dublin Animal Research Ethics committee, approval P-42-05, and licensed by the Department of Health & Children, Ireland, license number B100/3682. All animal protocols were conducted according to Institution guidelines for animal husbandry and welfare.

### Bacteria

Low passage isolates of *Leptospira interrogans* serovar Copenhageni strain RJ16441 were passaged through guinea pigs to maintain virulence as previously described [11]. Cultures were maintained at 30°C in Ellinghausen-McCullough-Johnson-Harris (EMJH) liquid medium (Becton Dickinson) supplemented with 6% rabbit serum (Sigma). Cultures were harvested at a density of 1×10^8^ leptospires/mL.

### Animals

Six male *Rattus norvegicus* Wistar strain (Charles River Laboratories, UK), 150–190 g, 6 weeks of age, were injected intraperitoneally with 5×10^7^ low passage *in vitro* cultivated *Leptospira* in a final volume of 500 µl. Rats were housed in metabolism cages once weekly and urine collected for enumeration of leptospires by darkfield microscopy, as previously described [8]. For DIGE analysis, urine samples were collected at 3 to 6 weeks post-infection as previously described [8]. Pellets were stored at −80°C until required. Rats were euthanized at 147 days post-infection; kidneys were removed and snap frozen in liquid nitrogen. Negative-control animals were injected with medium alone. A second group of rats were similarly infected in order to collect urine for analysis of immunoglobulin content.

### DIGE Sample preparation

In vitro cultivated leptospires (IVCL) were prepared as previously described [12]. In brief, after enumeration by dark-field microscopy, samples were harvested by centrifugation at 12,000 g for 10 min at 4°C and washed twice with ice-cold 10 mM Tris-1 mM EDTA. IVCL and rat urine derived samples were solubilised in lysis buffer (7 M urea, 2 M thiourea, 1% ASB-14) and stored at –20°C. For preparation of negative control urine spiked with IVCL, urine pellets were resuspended in lysis buffer and sufficient numbers of solubilised IVCL were added to emulate *Leptospira* numbers in infected urine samples (∼5×10^7^ *Leptospira*/mL urine). Protein concentrations were determined using the RC/DC protein assay kit (Bio-Rad).

### Protein-cyanine dye labeling

Protein samples were fluorescently labelled using CyDye DIGE Fluors (Amersham) as per manufacturer's instructions in order to compare samples derived from infected rats with samples from non-infected controls which were spiked with in vitro cultivated *Leptospira* (Table 1). For each gel, 50 µg of infected or non-infected spiked urine were added to 400 pmol (1 µL) of Cy3 or Cy5, and allowed to incubate on ice for 30 min. For each gel, 50 µg of pooled internal standard comprising equal µg amounts of infected and negative control samples were labelled with Cy2. The labelling reaction was quenched by the addition of 1 µL of 10 mM lysine for 10 min. During all stages of the experiment, samples were protected from light to prevent degradation of the CyDye labels.

**Table 1:** Experimental design for DIGE experiment: A) pH 3–10 or B) pH 4–7.

A	Gel #	Cy3	Cy2	Cy5
	1	Rat 7 (Neg)	Internal Control	Rat 1 (Inf)
	2	Rat 2 (Inf)	Internal Control	Rat 8 (Neg)
	3	Rat 9 (Neg)	Internal Control	Rat 3 (Inf)
	4	Rat 4 (Inf)	Internal Control	Rat 10 (Neg)
	5	Rat 9 (Neg)	Internal Control	Rat 5 (Inf)
	6	Rat 6 (Inf)	Internal Control	Rat 10 (Neg)

B	Gel #	Cy3	Cy2	Cy5
	1	Rat 7 (Neg)	Internal Control	Rat 1 (Inf)
	2	Rat 2 (Inf)	Internal Control	Rat 8 (Neg)
	3	Rat 9 (Neg)	Internal Control	Rat 3 (Inf)
	4	Rat 4 (Inf)	Internal Control	Rat 10 (Neg)
	5	Rat 9 (Neg)	Internal Control	Rat 5 (Inf)

Rat numbers 1, 2, 3, 4, 5 and 6 are experimentally infected (Inf) and shedding leptospires in urine whilst rat numbers 7, 8, 9, 10 are negative controls (Neg) which are spiked with in vitro cultivated leptospires. Each gel contains an infected and negative sample labeled with either Cy3 or Cy5, and an internal control with both samples labeled with Cy2.

### 2D gel electrophoresis

18 cm IPG strips were rehydrated overnight at room temperature with labelled proteins (Table 1) and proteins separated as previously described [12], [13].

### Image analysis

Gels were scanned using the Typhoon™ fluorescence gel scanner (Amersham). Different band-pass filters (520 nm for Cy2, 580 nm for Cy3 and 670 nm for Cy5) were used to image each of the three CyDyes. Differential protein expression was analyzed by Progenesis™ software (Amersham) following the software's manual. The 2D image of the gel from each infected rat sample was compared with that of the negative spiked urine sample via the pooled internal standard. Volumes representing the sum of pixel intensities within the spots were normalized with the total spot volume from the pooled internal standard. All fold differences were based on normalized spot volumes. To excise and identify differentially expressed protein spots, 250 µg of the unlabelled pooled internal standard was separated by 2D SDS-PAGE as described above and stained using Sypro Ruby (Sigma). Spots of interest were picked using a 3.0 mm spot picker (The Gel Company) and stored at −20°C until analysis.

### Nano-liquid chromatography with data-dependent tandem mass spectrometry (nLC-MSMS)

Excised gel spots were processed as previously described [8]. Eluted peptides were analyzed by nLC-MSMS using data-dependent acquisition mode on a hybrid linear ion-trap Fourier-transform ion cyclotron resonance spectrometer (7 Telsa LTQ FT Ultra, Thermo Scientific, Bremen, Germany) operated with nano-electrospray ionization in positive ion mode. After dissolution in 10 µL 0.1% formic acid, 1% acetonitrile (v/v) samples were injected onto a trapping column (3 cm, 100 µM, C18, Micro-Tech) previously equilibrated in 100% A (A, 0.1% formic acid, 1% acetonitrile in water; B, 0.1% formic acid in acetonitrile) at a flow rate of 2 µL/minute. Following 10 minutes washing, the trapping column was eluted through a pre-equilibrated analytical column (15 cm, 75 µM, C18, Micro-Tech) at a flow rate of 300 nL/minute using a compound linear gradient (3 min at 95% A; 85% A, 15% B at 8 min; 65% A, 35% B at 18 min; 25% A, 75% B at 30 min and 90% A, 10% B at 50 min). Column eluent was directed to an uncoated pulled silica nanospray tip (Picotip FS360-20-10-N-5-C12, New Objective) at 2.4kV for ionization without nebulizer gas. The mass spectrometer was operated in data-dependent mode with a precursor survey scan (350–2000 m/z) at 100,000 resolution (at m/z 400), and data-dependent MSMS in the ion trap for the top 6 precursor ions while employing optimized dynamic exclusion settings in recruiting those ions.

### Database search

Resulting ion spectra were interpreted using Mascot software (http://www.matrixscience.com; Matrix Sciences, London, United Kingdom). Peptide sequences were first matched against a *Leptospira*/Rat database comprising *L. interrogans* serovar Copenhageni strain Fiocruz L1-130 sequence database (AE016824/AE016823) and *Rattus norvegicus* sequence database (NCBInr 20090430) downloaded from NCBI (www.ncbi.nlm.nih.gov), using the Mascot software. Peptide sequences were also used to search the NCBI non-redundant database. Database search parameters included a peptide mass tolerance of ±0.5Da, fragment mass tolerance of ±0.8 Da, Carbamidomethyl (C) fixed modifications and Oxidation (M) variable modifications. MS/MS spectra matched to peptide sequences exceeding p-value of 0.05 were examined manually, specifically with respect to calculated parent and product ion mass accuracy as well as to whether the result was fully or partially tryptic.

### RNA

Samples for RNA extraction were thawed on ice and 1 ml TRI reagent (Sigma) was added. Tissue samples were diced and incubated on ice for 15 min and centrifuged at 4000 g to remove excess tissue. To the Tri-reagent mixture, 200 µL of 100% chloroform (Sigma) was added and tubes were inverted several times. The mixture was allowed to incubate for 10 min on ice. Samples were then centrifuged at 21,000 g for 15 min. The aqueous phase was carefully removed to avoid the white fluffy layer and total RNA was precipitated with two volumes 100% ice cold iso-propanol (Sigma) overnight at −20°C. Samples were centrifuged at 21,000 g at 4°C for 35 min. The resulting pellets were washed twice with 70% ethanol. Samples were allowed to air dry for 10 min, and were resuspended in 100 µL of nuclease free water. RNA quality and quantity was determined by fluorospectrometer (Nanodrop ND 1000, Coleman Technologies, V3.5.2) and a 2100 Bioanalyser (Agilent Technologies). Samples with an RNA integrity number of greater than or equal to 8 and a 28S/18S rRNA ratio of 1.7–2.1 were used in all experiments.

### Quantitative reverse-transcriptase PCR (qRT-PCR)

RNA (up to 5 µg) was treated with 1 U of DNase I (Qiagen) in 2x buffer for 15 min at room temperature. Subsequently, 1 µg RNA was reverse transcribed using the First Strand Synthesis kit (Invitrogen), as per manufacturer's instructions, with 1 µL of oligo dT, 10 µM dNTPs, 10× RT buffer, 25 mM MgCl_2_, 0.1 M DTT, 40 U/µL of RNase out and 10 U/µL superscript III. Primers (Table 2) were designed using primer3 (http://frodo.wi.mit.edu/primer3/). Primers were diluted to 5 pmol/µL in ddH_2_O. Each reaction comprised 1 µL of cDNA, 1 µL of each primer, 12.5 µL 2xPCR mix (Sybr green dye, dNTPs, Taq, MgCl_2_ (ABI)) and 9.5 µL of ddH_2_O. No template controls were added to generate negative controls. Each reaction was carried out in duplicate. Samples were placed on a 96 well plate, sealed and centrifuged at 1000 g for 1 min. The plate was run in an Applied Biosystems 7300 RealTime machine for 40 cycles (95°C 10 min, 95°C 1 min 60°C 1 min). A dissociation curve step was added (45–95°C) to ensure optimization of the primers. CT values were calculated at a cut-off of 0.05. Relative expression of transcripts was calculated using the 2^-ΔΔCT^ method [14]. Two tail student t-tests were performed to determine significance of transcript expression values, and error determined using standard error of the mean.

**Table 2:** List of primers used for quantitative reverse-transcriptase PCR.

Primer Name	Sequence	Tm°C	Product Size (bp)	Gene
*mme*_fwd	AGCTGAAGAGAAGGCCCTGGCA	64	226	NM_012608.2
*mme*_rev	ATTGACTACCGCCGCGCCAC	63.5	226	NM_012608.2
*mdh1*_fwd	CGACTGTGCAGCAGCGTGGT	63.5	131	NM_033235.1
*mdh1*_rev	CGACACGAACTCGCCCTCCG	65.5	131	NM_033235.1
*igκ*_fwd	CCTGGCAGGTCTCCGAAGCG	65.5	223	BC062802.1
*igκ*_rev	TTGGTGCAGCATCAGCCCGT	61.4	223	BC062802.1
*igα*_fwd	GCCAGCTGCAGAGTGCCCAA	63.5	126	AJ5110151.1
*igα*_rev	AGGCGAGGGCGGCAGACTAA	63.5	126	AJ5110151.1
*napsa*_fwd	CTAGGACCCCCACCTCCGGC	67.6	193	NM_031670.2
*napsa*_rev	TGGTGGAACCAGCAGGCCAA	61.4	193	NM_031670.2
*β-actin*_fwd	GCGTCCACCCGCGAGTACAA	59.4	122	NM_031144.2
*β -actin*_rev	TTGCACATGCCGGAGCCGTT	59.9	122	NM_031144.2

### Immunoblotting

In vitro cultivated leptospires were separated by 1-D gel electrophoresis and transferred to PVDF as previously described [8]. Membranes were probed directly with undiluted urine overnight at 4°C, followed by incubation with horseradish peroxidise-goat anti-rat IgG conjugate (1∶2500) (Sigma). Bound conjugates were detected with SuperSignal WestPico (Pierce) and images acquired using a UVP Biospectrum – AC w/Bio Chemi Camera (Cambridge, UK).

## Results

### Chronic infection of rats with *L. interrogans* serovar Copenhageni

By 7 days post-infection, experimentally infected rats shed detectable numbers of leptospires in urine, Table 3. By 21 days post-infection, six rats were shedding at least 10^7^ leptospires/ml of urine.

**Table 3:** Numbers of *Leptospira* excreted per mL rat urine following experimental infection.

Rat #	Week 0	Week 1	Week 2	Week 3	Week 4	Week 5	Week 6
**1**	0	0	1.0E+5	1.0E+7	9.0E+6	3.6E+6	5.6E+6
**2**	0	1.0E+5	1.0E+5	5.0E+7	1.5E+7	8.8E+6	3.2E+6
**3**	0	0	0	1.0E+7	6.0E+6	7.4E+6	6.4E+6
**4**	0	2.0E+5	0	2.0E+7	6.6E+6	8.2E+6	1.1E+7
**5**	0	4.0E+5	9.0E+5	3.0E+7	3.0E+7	1.4E+7	1.6E+7
**6**	0	0	0	2.0E+8	1.5E+7	1.0E+7	4.6E+6

### 2-dimensional difference gel electrophoresis (2-D DIGE)

Urine samples from experimentally infected and non-infected control rats were analysed by 2-D DIGE to identify differentially expressed proteins. Samples were included from week 3 to week 6 post-infection to reduce effects of day-to-day biological variation and identify only the most robust differences. In vitro cultivated *Leptospira* (IVCL) were added to negative control urine samples to facilitate a comparison of the proteome of IVCL against that of leptospires excreted in rat urine, in addition to a comparison of the proteome of urine pellets from experimentally infected rats against that of negative controls.

**Figure 1:**
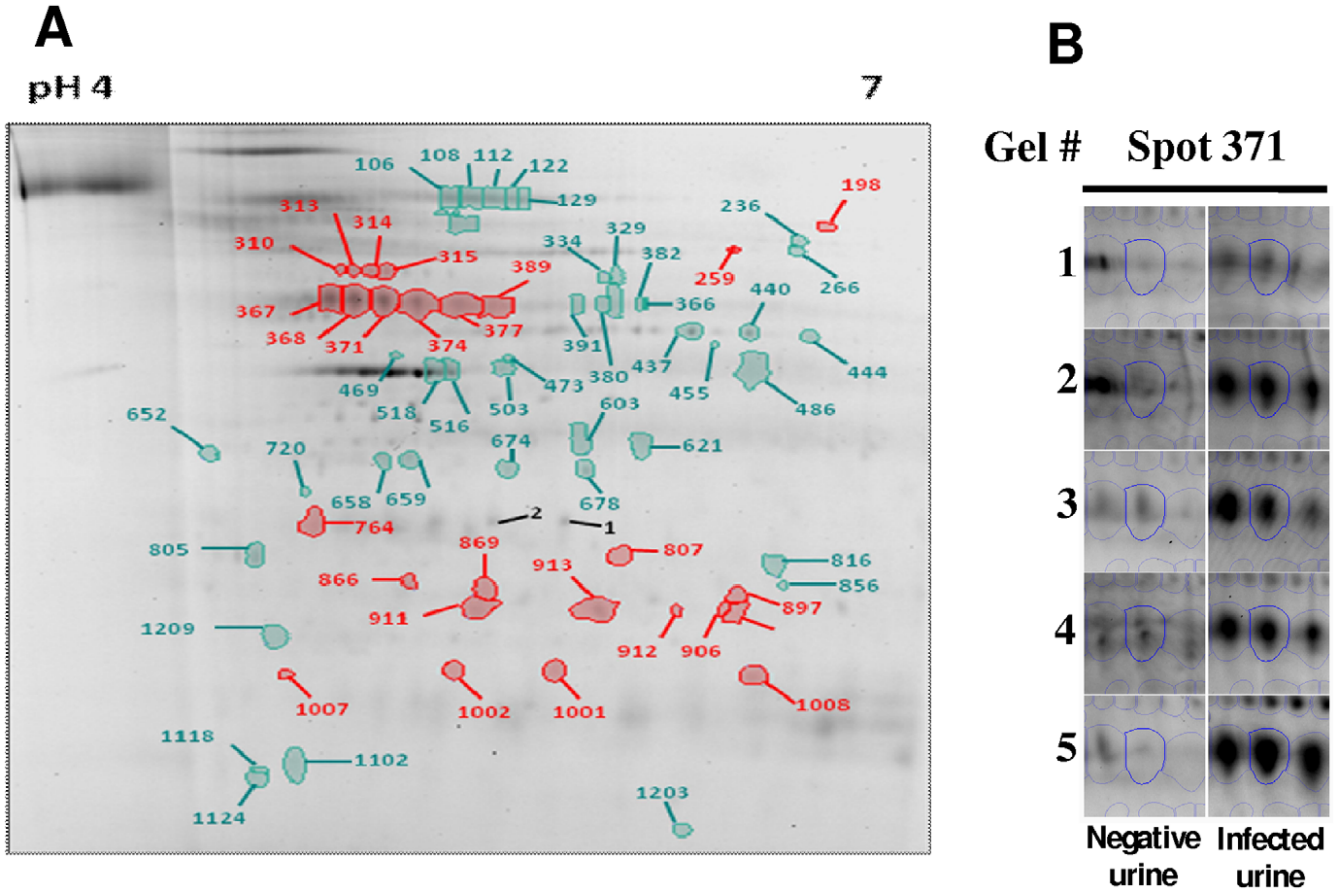
2D DIGE. **A**) Spots numbered in red are increased in the urine of rats chronically infected with *L. interrogans*. Spots numbered in green are increased in negative control urine spiked with in vitro cultivated *Leptospira*. Spot numbers correlate to those listed in Table 4 and Table S1. **B**) Image of each gel replicate highlighting spot 371 which is increased in infected rat urine compared to the negative control.

Analysis of urine pellets by DIGE separated over pH 3–10 aligned a total of 1029 proteins, 17 of which were differentially expressed (p<0.05, power >0.8) (data not shown); 10 spots were upregulated in infected rat urine whilst 7 were upregulated in non-infected control samples. Analysis of urine pellets separated over pH 4–7 aligned a total of 1209 proteins, 25 of which were differentially expressed (p<0.05, power >0.8); 16 spots were upregulated in infected rat urine whilst 9 were upregulated in control samples. Fold changes ranged from 1.3 to 3.9. Forty four additional differentially expressed proteins were detected when the power was reduced, Figure 1, as indicated below.

### Identification of differentially expressed proteins

Differentially expressed protein spots were excised from protein gels separated over pH 4–7 for identification by mass spectrometry. Whilst the majority of identified proteins were from *Rattus norvegicus*, several proteins of *L. interrogans* were identified, Table 4.

**Table 4:** Differentially expressed proteins identified by mass spectrometry.

Spot^a^	Accession^b^	Annotation^c^	Coverage^d^	Mascot^e^	Fc^f^
***R. norvegicus***					
106, 108, 112, 122,	GI:6981210	Membrane metallo endopeptidase	23, 9, 17, 30	656, 101, 462, 595	-1.5, -1.7, -1.7, -1.9
603, 621	GI:15100179	Malate dehydrogenase 1, NAD (soluble)	2, 7	34, 70	-1.3, -1.3
503, 516, 518	GI:13928928	Napsin A aspartic peptidase	2, 11, 8	40, 113, 149	-1.8, -1.5, -1.6
658, 659, 674	GI:57526957	Aspartoacylase	14, 8, 5	98, 83, 38	-1.3, -1.4, -1.5
367, 368, 371, 374, 377, 389	GI:17105370	Vacuolar H+ATPaseB2	1, 8, 1, 1, 31	48, 133, 70, 51, 713	+1.6, +1.9, +2.8, +2.6, +1.7, +1.3
367, 368, 377, 389	GI:13591914	Kidney aminopeptidase M	2, 2, 1, 1	80, 201, 69, 102	+1.6, +1.9, +1.7, +1.3
371, 374	GI:23559227	Similar to Ig heavy chain	11, 4	156, 67	+2.8, +2.6
*367, 368, 371, 374, 389	GI:23559227	Ig alpha heavy chain	6, 6, 11, 9, 3	119, 111, 285, 155, 61	+1.6, +1.9, 2.8, +2.6, +1.3
*911, 913	GI:109157157	Chain L, Cd8alpha- Alpha in complex with Yts 105.18 Fab	16, 16	225, 144	+3.9, +2.6
259	GI:6978809	ATP synthase	4	58	+1.3
***Leptospira***					
310,313,314,315	GI:45600451	GroEL	52, 28, 14,29	1126, 690, 493, 476	+1.8, +1.9, +2.1, +1.7
1008	GI:45599329	Loa22	14	131	+2.1
1, 2	GI:45600468	LipL32	18, 4	296, 96	0
652	GI:45600643	flagellin	45	866	-2.3

a Spot numbers correlate with numbers in Figure 1A.

b Accession number from NCBI.

c Protein annotation.

d Percentage of protein coverage.

e Mascot score.

f Fold change relative to infected samples.

*Additional hits were identified when searched against the non redundant database.

Differentially expressed proteins derived from *Rattus norvegicus* included spot numbers 367, 368, 371, 374, 377 and 389 that were increased 1.6, 1.9, 2.8, 2.6, 1.7 and 1.3 fold respectively (range of p values = 0.00060–0.03565, power = 0.601–0.997) and contained multiple protein species. Each contained vacuolar H+ATPase B2, whilst spot numbers 367, 368, 377 and 389 also contained kidney aminopeptidase, and spot numbers 371 and 374 also contained immunoglobulin heavy chains. Spot numbers 911 and 913 were increased 3.9 (p = 0.0003, power = 1) and 2.6 (p = 0.00015, power = 1) fold respectively and were identified as containing immunoglobulin light chain. Spot number 259, increased 1.3 fold in infected urine, was identified as ATP synthase.

Spot numbers 106, 108, 112 and 122 were increased 1.5, 1.7, 1.7 and 1.9 fold respectively in negative control samples (range of p values = 0.02288–0.04364, power = 0.547–0.693) and identified as membrane metallo endopeptidase. Similarly spot numbers 603 and 621 were increased in negative control samples 1.3 fold (p = 0.01181 and 0.2357, power = 0.81 and 0.687) and identified as malate dehyrogenase. Spot number 503 was increased 1.8 fold (p = 0.03564, power = 0.602) in negative control urine and was identified as napsin A aspartic peptidase. Spot numbers 516 and 518, which were increased 1.5 (p = 0.04934, power = 0.349) and 1.6 (p = 0.04770, power = 0.535) fold respectively in negative urine, were also identified as napsin A aspartic peptidase. Finally, spot numbers 674, 659 and 658 were also increased in negative control samples 1.5, 1.4 and 1.3 fold respectively (range of p values = 0.00419–0.04346, power = 0.557–0.932) and identified as aspartoacyclase-3. Table S1 provides Mascot-matched peptide sequences used for protein identification.

Differentially expressed proteins were identified from *Leptospira interrogans*. Spot numbers 310, 313, 314 and 315 were identified as GroEL and were increased in infected urine samples 1.8, 2.1, 1.9 and 1.7 fold respectively (range of p values = 0.00149–0.04736, power = 0.539–0.984) compared to the negative control containing in vitro cultivated *Leptospira*. Spot number 1008 was increased 2.1 fold (p = 0.02975, power = 0.639) in infected urine samples and was identified as Loa22, an outer membrane lipoprotein which contains an OmpA domain and the first known virulence-associated gene of pathogenic *Leptospira* species. In contrast, spot number 652 was increased 2.3 fold (p = 0.01061, power = 0.826) in negative control samples compared to infected urine samples, and was identified as a leptospiral flagellin protein. As an added control, two additional spots (number 1 and 2) which were not differentially expressed, were excised and identified as the outer membrane lipoprotein LipL32. Table S1 provides Mascot-matched peptide sequences used for protein identification.

### Quantitative reverse-transcriptase PCR (qRT-PCR) during chronic leptospirosis

**Figure 2:**
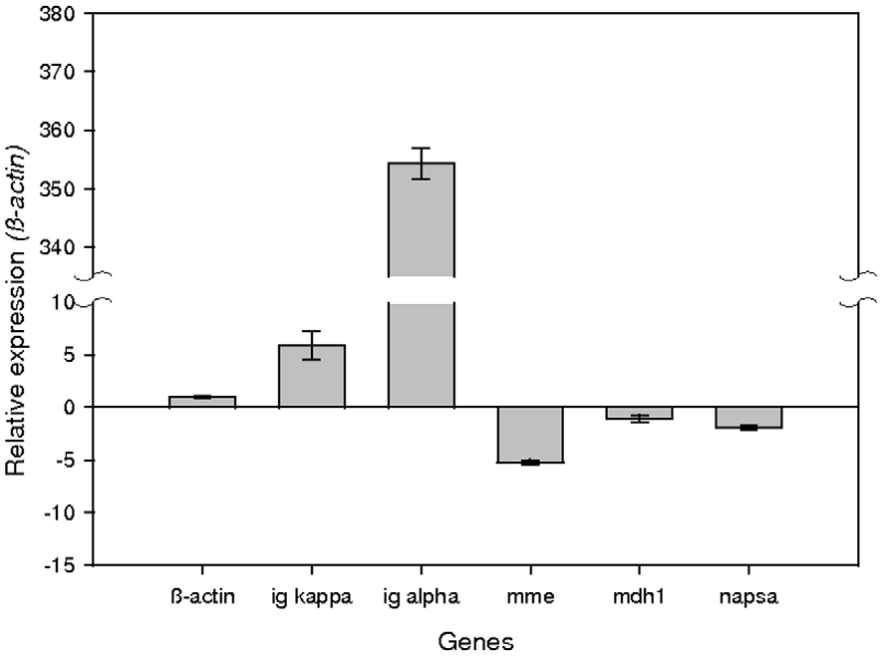
Relative mRNA expression values of genes in the kidney of experimentally infected *Rattus norvegicus*. Gene expression values in experimentally kidney tissues (N = 4) were normalized to the expression of the reference gene β-actin and compared to negative control renal tissues (N = 4). Error bars represent SEM.

Kidney tissue was assessed by qRT-PCR for differential expression of host genes, Figure 2. Immunoglobulin light chain kappa (*igκ*) and immunoglobulin heavy chain α (*igα*) were up-regulated 5.90±1.35 (p = 0.049) and 354.29±2.54 (p = 0.000125) fold relative to the reference gene *β-actin* [15] in four infected animals compared to 4 healthy controls. In contrast, and in general agreement with protein expression data derived from urine samples, membrane metal endopeptidase (*mme*) was downregulated -5.24±0.24 fold (p = 0.0000238) in infected kidney compared to negative controls. The gene encoding malate dehydrogenase, *mdh1*, was not differentially expressed (-1.08±0.34) whilst the gene encoding napsin A aspartic peptidase, *napsa*, showed decreased expression, -1.91±0.2 (p = 0.0002), in infected kidney compared to non-infected controls.

### Urine derived immunoglobulin

**Figure 3:**
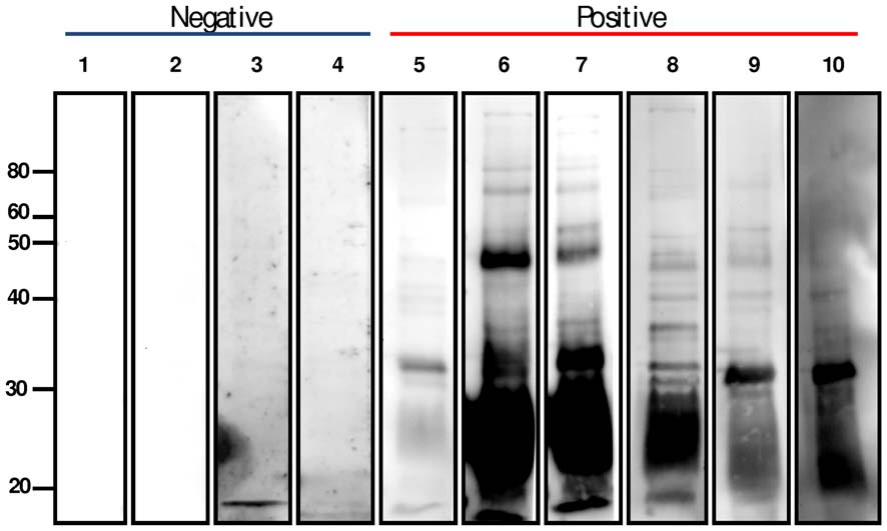
Immunoblotting of in vitro cultivated leptospires (IVCL). 10^7^ IVCL/lane were separated by 1-D gel electrophoresis and probed with urine collected at 12 weeks post-infection from experimentally infected rats and non infected controls. Molecular mass markers are indicated on left.

Since urine pellets derived from infected rats contained increased levels of immunoglobulin compared to negative controls as determined by DIGE and qRT-PCR, immunoblots were performed to validate 2-D DIGE and qRT-PCR data and determine whether immunoglobulin in urine from infected rats was specific for leptospires, Figure 3. Immunoblotting with urine from infected rats contained IgG which was specific for IVCL. No specific reactivity was detected using urine from non-infected controls.

## Discussion

*Rattus norvegicus* is a significant reservoir host of leptospirosis. It is also an important experimental animal model making it ideal to study pathogenic mechanisms of chronic leptospirosis using a relevant host of persistent carriage and infection. Recent studies have confirmed that *Leptospira* regulate gene and protein expression during acute and chronic disease [8], [12], [16] and whilst several studies have examined the acutely infected host response to infection, there has been limited work to explore the molecular basis of the chronically infected host response that facilitates persistent carriage.

Experimentally infected *Rattus norvegicus* excrete large numbers of leptospires and differential protein expression is evident in the urine of experimentally infected rats compared to non-infected controls [8]. In order to normalize for the presence of leptospires in urine from infected rats, urine from non-infected controls was spiked with in vitro cultivated *Leptospira*. Therefore, results identify not only host derived proteins that are differentially expressed during chronic leptospirosis, but also those proteins of *Leptospira* that are differentially expressed in response to host signals encountered during renal colonization.

The expression of several host derived proteins was diminished in infected rat urine samples relative to non-infected controls and included membrane metalloendopeptidase (Mme), malate dehydrogenase 1 (Mdh1), napsin A aspartic peptidase (Napsin A) and aspartocylase. Membrane metalloendopeptidase (also known as neprilysin), a type-II membrane anchored enzyme, has roles in posttranslational modification, protein turnover, and as a chaperone. Napsin A aspartic peptidase is a kidney-derived aspartic protease-like protein expressed in kidney, lung and spleen, and is excreted as a functional protease in urine [17], [18]. Whilst the significance of this reduced expression during chronic leptospirosis is not yet clear, both Mme and Napsin A are reported to be expressed in renal tubules, and decreased levels of expression are indicative of renal tubule injury [17], [19]. Alternatively, *Leptospira* might directly affect the expression of these host proteins as a protective mechanism to minimize host-initiated proteolytic degradation of leptospiral proteins. Primary injury of renal tubules is regarded as the hallmark of the kidney in human patients suffering leptospirosis [20], and both Mme and Napsin A are conserved in humans and dogs.

In contrast, the expression of host derived proteins identified as components of immunoglobulin G and A, vacuolar H+ATPase B2 and kidney aminopeptidase, were identified in protein spots detected in increased amounts in urine of chronically infected rats. Vacuolar H^+^-ATPases mediate the ATP-dependent transport of protons, are expressed in the plasma membrane in the kidney and contribute to proximal tubular bicarbonate reabsorption. The importance in final urinary acidification along the collecting system is highlighted by monogenic defects in two subunits of the vacuolar H^+^-ATPase in patients with distal renal tubular acidosis [21]. Aminopeptidases are proteolytic enzymes that remove L-amino acids sequentially from the amino termini of polypeptide chains. Both vacuolar H+ATPase and kidney aminopeptidase have been identified in the urinary proteome of rats [22].

Since experimentally infected rats can persistently excrete leptospires for months [23], samples taken over a three-week period were selected for DIGE to eliminate day-to-day variability in urine samples. However, continued evidence of differential gene expression in infected kidney tissue compared to non-infected controls was provided by qRT-PCR at the end-point of the experiment. In contrast to gene expression levels for *igk* and *igα* which were significantly upregulated, decreased level of *mme* and *napsa* gene transcripts were detected which is in general agreement with proteomic data and indicative of differential protein expression. However, it will be important to ascertain at exactly what time post infection differential proteomic and gene expression changes occur, and whether such changes are linked to the appearance of pathology.

Our results indicate that increased amounts of Loa22, a surface exposed putative lipoprotein, is expressed by leptospires excreted in urine from chronically infected rats compared to in vitro cultures. Similarly, increased amounts of multiple isoforms of GroEL are detected. Loa22 is the first genetically defined virulence factor in pathogenic *Leptospira* species since mutation of the gene encoding *loa22* results in attenuation of virulence [24]. In addition, expression of Loa22 is upregulated during acute disease relative to other outer membrane proteins [12]. Increased expression of GroEL can be induced by modifying growth temperature of cultures from 30 to 37°C overnight, but interestingly there is no corresponding increase in gene transcript [25]. LipL32 is an outer membrane lipoprotein which is expressed during acute and chronic disease, the function of which remains to be elucidated [8], [12], [26]. Paradoxically, LipL32 is a major outer membrane protein specific to pathogenic species of *Leptospira*, yet mutations in this gene do not confer any change in phenotype including virulence [27]. Although LipL32 is detected in significant amounts in leptospires excreted in urine, differential expression was not detected for those isoforms excised for identification. Decreased amounts of flagellin were detected in leptospires excreted in urine; this may reflect diminished motility during renal tubule colonization, and that motility increases when leptospires are in the external environment. A similar diminution in levels of flagellin was observed when leptospires were cultured at 37°C from 30°C in order to emulate host conditions encountered during infection [28].

To further verify that the increased immunoglobulin content in urine of infected rats was in response to infection, immunoblots were performed using in vitro cultivated leptospires. Immunoblots were probed directly with urine from infected rats compared to urine from non-infected controls for the presence of IgG. Urinary IgG from infected rats was specific for leptospires and reacted with several protein antigens. In previous studies, it has been shown that sera from chronically infected rats reacts with LipL32 as expressed by both leptospires excreted in urine and the in vitro cultivated leptospires with which rats were experimentally infected, but relatively few other antigens [8]. This suggests that antigen expression was down-regulated by leptospires in renal tubules to avoid host antibody responses; it will be interesting to determine the specificity of urinary IgG compared to serum IgG, particularly for antigens expressed by leptospires excreted in urine. During bovine leptospirosis, there is a correlation between the rise in urinary agglutinating antibody levels and a reduction in the detection of viable leptospires present in urine [29]. Plasma cells associated with tubulointerstitial nephritis in dogs have been shown to produce anti-leptospiral antibody locally [30], but no anti-kidney antibody had been detected in any of the renal eluates, suggesting that antibody production is directed specifically against *Leptospira* and not against renal antigens [30]. The identification of protein antigens reactive with urinary IgG can also provide for the development of novel diagnostics to detect reservoir hosts of infection, including humans [5].

Quantitative RT-PCR suggests increased expression of IgA in addition to IgG. Although immunoblots confirmed detection of IgG specific for leptospires, a corresponding detection of IgA specific for leptospires was not detected in urine (data not shown). This may be due to the different time point of sampling or alternatively, indicative of local production of IgA which is not excreted in urine. Our analysis at this time is limited to pellets of urine from infected rats which contain excreted leptospires as well as host derived proteins. However, it is likely that analysis of the supernatant will provide for the identification of additional host derived proteins. It will be of interest to further investigate the supernatant for biomarkers of chronic leptospirosis and monitor relative expression of these in the presence of urine over the course of months, as occurs in naturally infected hosts suffering chronic disease. By definition of the experimental design, differentially expressed proteins serve as biomarkers to identify chronically infected rats compared to non-infected controls, but such biomarkers will need to be validated in hosts with alternative kidney pathologies e.g. chronic kidney disease. Finally, it will be important in future studies to determine what components of *Leptospira* induce increased/decreased expression of each of these host derived proteins and whether inhibiting such changes reduces colonization.

In conclusion, results demonstrate the use of the rat model of chronic leptospirosis to identify differentially expressed proteins in urine derived from both host and pathogen. Differentially expressed host derived proteins include known markers of kidney function and immune response. Differential expression was validated at the level of gene transcription and in the case of immunoglobulin G, further validated through to the production of antibody which was specific for leptospires. Differentially expressed pathogen derived proteins include the known virulence factor Loa22 and the stress response protein GroEL.

## Supporting Information

Table S1 Mascot-matched peptide sequences used for protein identification.
(XLSX)
